# Inhibitors of mitogen-activated protein kinases differentially regulate costimulated T cell cytokine production and mouse airway eosinophilia

**DOI:** 10.1186/1465-9921-6-36

**Published:** 2005-04-15

**Authors:** Ligia Chialda, Meixia Zhang, Kay Brune, Andreas Pahl

**Affiliations:** 1 Department of Experimental and Clinical Pharmacology and Toxicology, University of Erlangen-Nürnberg, Fahrstr. 17, D-91054 Erlangen, Germany; 2 Present Address Department of Clinical Pharmacology, Chinese Medical University, Shenyang, China

## Abstract

**Background:**

T cells play a dominant role in the pathogenesis of asthma. Costimulation of T cells is necessary to fully activate them. An inducible costimulator (ICOS) of T cells is predominantly expressed on Th2 cells. Therefore, interference of signaling pathways precipitated by ICOS may present new therapeutic options for Th2 dominated diseases such as asthma. However, these signaling pathways are poorly characterized *in vitro *and *in vivo*.

**Methods:**

Human primary CD4^+ ^T cells from blood were activated by beads with defined combinations of surface receptor stimulating antibodies and costimulatory receptor ligands. Real-time RT-PCR was used for measuring the production of cytokines from activated T cells. Activation of mitogen activated protein kinase (MAPK) signaling pathways leading to cytokine synthesis were investigated by western blot analysis and by specific inhibitors. The effect of inhibitors *in vivo *was tested in a murine asthma model of late phase eosinophilia. Lung inflammation was assessed by differential cell count of the bronchoalveolar lavage, determination of serum IgE and lung histology.

**Results:**

We showed *in vitro *that ICOS and CD28 are stimulatory members of an expanding family of co-receptors, whereas PD1 ligands failed to co-stimulate T cells. ICOS and CD28 activated different MAPK signaling cascades necessary for cytokine activation. By means of specific inhibitors we showed that p38 and ERK act downstream of CD28 and that ERK and JNK act downstream of ICOS leading to the induction of various T cell derived cytokines. Using a murine asthma model of late phase eosinophilia, we demonstrated that the ERK inhibitor U0126 and the JNK inhibitor SP600125 inhibited lung inflammation *in vivo*. This inhibition correlated with the inhibition of Th2 cytokines in the BAL fluid. Despite acting on different signaling cascades, we could not detect synergistic action of any combination of MAPK inhibitors. In contrast, we found that the p38 inhibitor SB203580 antagonizes the action of the ERK inhibitor U0126 *in vitro *and *in vivo*.

**Conclusion:**

These results demonstrate that the MAPKs ERK and JNK may be suitable targets for anti-inflammatory therapy of asthma, whereas inhibition of p38 seems to be an unlikely target.

## Background

Asthma is a chronic inflammatory disease of the airways. The inflammation characterizing asthma is complex and involves multiple cells and mediators. The cells involved include well-recognized immune and inflammatory cells, lymphocytes, macrophages, eosinophils, mast cells and neutrophils as well as resident lung cells [1,2]. The importance of allergen-specific CD4^+ ^Th2 cells has been demonstrated. Th2-associated cytokines such as IL-4, IL-5, IL-9 and IL-13 are known to be involved in IgE production, airway eosinophilia, and airway hyperresponsiveness [3]. Consequently, the inhibition or modulation of allergen-specific Th2 cells and their cytokines has become an attractive target for novel therapeutic intervention strategies [4-6]. Downregulation of cytokine production has been achieved by glucocorticoids and immunosuppressants both *in vitro *and *in vivo*. As these agents suppressed a broad spectrum of immune function, more specific regulatory pathways of T cell need to be addressed.

In recent years, considerable effort has been mounted to dissect the signaling events in T cells. Full activation of T cells requires signaling through both the TCR/CD3 complex and the CD28 costimulatory receptor [7-10]. CD28 engagement by B7-1 and B7-2 on resting T cells provides a primary costimulatory signal critical for initial cell cycle progression, interleukin 2 production and clonal expansion [11]. Engagement of CTLA-4 by the same B7-1 or B7-2 ligands results in attenuation of T cell responses. Recently, molecular homologues of CD28 and CTLA-4 coreceptors and their B7-like ligands have been identified. These homologues presumably play an essential role in the acquisition of effector function and/or tolerance induction. One of the CD28-like molecules is induced during activation of T cells, thus it is referred as an inducible costimulator (ICOS) and has a unique B7-like ligand (B7-H2, B7 h or B7RP-1). PD-1 is an inhibitory CD28-like receptor, with two B7-like ligands (PD-L1 and PD-L2) [12,13]. It has been shown that the ICOS/B7-H2 pathway controls T cell dependent immune responses [14-17].

Several protein kinases such as the family of MAPKs are involved in the transmission of extracellular signals into the nucleus. [18]. MAPKs are serine/threonine kinases that include extracellular signal-regulated kinases (ERKs) [19], Jun NH2-terminal kinases (JNKs) [20] and p38 MAPK [21]. CD3 signaling alone has been shown to activate ERK, and the combination of CD3 and CD28 signaling can synergistically activate JNK in T cell lines and clones [22,23]. In these studies, CD28 signaling alone was shown to minimally activate ERK and JNK, while others have reported much more significant ERK activation [24]. Activation of p38 has also been observed in Jurkat cells and in mouse T cells treated with CD3/CD28 [25,26]. While it has become clear that productive T cell activation requires the cooperation of multiple signaling pathways, the composition and the relative contribution of these pathways to the activation process and cytokine production remain yet to be fully deciphered.

The predominant expression of ICOS on Th2 cells and its effect on the development of Th_2 _cells has been reported [15,27]. Therefore, interference of signaling pathways precipitated by ICOS may present new therapeutic options for Th2 dominated diseases such as asthma. However, these signaling pathways are poorly characterized. Recently, we reported that the ERK pathway plays a pivotal role in regulating IL-13 production in human CD4^+ ^T cells after CD28 costimulation [28]. In the present study, we studied the activation of different MAP kinases in human CD4^+ ^T cells stimulated by α-CD3/α-ICOS, their function in the production of cytokines from these cells and compared these results to α-CD3/α-CD28 stimulated T cells. Our results demonstrate, that in human CD4^+ ^T cells, p42/p44 ERK, p38 and JNK differentially regulate the production of IL-2, IL-4, IL-13 and IFN-γ in response to α-CD3/α-CD28 or α-CD3/α-ICOS. Furthermore, inhibitors able to inhibit ICOS induced signaling proved to be effective in the murine asthma model of late phase eosinophilia.

## Methods

### Reagents

Oligonucleotides were synthesized by TIB Molbiol (Berlin, Germany). DMSO, TPA, ionomycin, dexamethasone, Histopaque-1077 was from Sigma Chemical Co (Deisenhofen, Germany). U0126, SP600125 and SB203580 were purchased from Biotrend Chemikalien GmbH (Köln, Germany). Purified anti-human CD3 and purified anti-human CD28 were from PharMingen Becton Dickinson Co (Heidelberg, Germany). RPMI 1640 medium was from Life Technologies (Heidelberg, Germany). Unless otherwise indicated, all other chemicals were purchased from the Sigma Chemical Co (Deisenhofen, Germany).

### Preparation of CD4^+ ^T cells

Buffy coats from healthy human volunteers were obtained from the Erlangen Blood Bank. PBMCs were isolated by density gradient centrifugation over Histopaque 1077 (Sigma, Deisenhofen, Germany), washed twice in Hanks buffer (Life Technologies, Heidelberg, Germany) and resuspended in RPMI 1640 medium (Roche Diagnostics, Penzberg, Germany). PBMCs were incubated with a hapten-antibody cocktail (containing monoclonal hapten-conjugated CD8, CD11b, CD16, CD19, CD36 and CD56 antibodies, Miltenyi Biotec, Bergisch Gladbach, Germany) and MACS anti-hapten microbeads (CD4^+ ^T cell isolation kit, Miltenyi Biotec, Bergisch Gladbach, Germany). CD4^+ ^T cells were isolated by negative selection on LS+ columns using a high gradient magnetic cell separation system MACS (Miltenyi Biotec, Bergisch Gladbach, Germany) according to the manufacturer's instructions. Purity of CD4^+ ^T cells was assessed by flow cytometry and was 90–95% (FACScan, Becton Dickinson, Heidelberg, Germany). Additional cells were monocytes (4–8%) and B cells (1–2%), which did not respond to α-CD3 antibodies. Purified CD4^+ ^T cells were resuspended in RPMI 1640 medium.

### Cell culture

For cytokine production, CD4^+ ^T cells were resuspended at 0.25 × 10^6 ^cells/ml and incubated in 500 μl aliquots in 24-well tissue culture plates (Falcon Becton Dickinson Labware, Heidelberg, Germany) at 37°C, 5%CO_2_. After preincubation with test substances for 30 min, cells were stimulated with α-CD3/α-CD28 Dynal beads (8*10^4^/2 μl/well). B7-H1, B7-H2 and B7-H3 binding by their receptors was assessed by stimulation of the cultured cells with self coated Dynabeads M-450 Epoxy (Dynal Biotech GmbH, Hamburg, Germany). α-CD3 mAb (0.1 μg/μl) (Pharmingen, Heidelberg, Germany) together with 0.04 μg/μl recombinant fusion proteins of B7-H1, B7-H2 and B7-H3 respectively, (R&D, Wiesbaden-Nordenstadt, Germany), were added to the prewashed beads according to the manufacturer's recommendations. The beads were added at a ratio of 3:1 to the CD4^+ ^T cells. At the indicated times (presented in figure legends), cells were sedimented by centrifugation, the supernatants were harvested and kept frozen at -80°C until cytokine protein determination; the cell pellet was lysed by RLT lysis buffer (Qiagen, Hilden, Germany) and frozen at -80°C until RNA isolation.

### Enzyme-linked immunosorbent assay

Cytokine measurements in culture supernatants were done by sandwich ELISA using matched antibody pairs (BD Pharmingen, Heidelberg, Germany). ELISA plates (Maxisorb, Nunc) were coated overnight with anti-cytokine mAb in 0.1 M carbonate buffer, pH 9.5. After being washed, plates were blocked with Assay Diluent (Pharmingen, Heidelberg, Germany) for 1 h and washed again. Appropriately diluted supernatant samples and standards were distributed in duplicates and the plates were incubated for 2 h at room temperature. Plates were washed, incubated for 1 h with working detector (biotinylated anti-cytokine Ab and Avidin-horseradish peroxidase conjugate). After washing, substrate (TMB and hydrogen peroxide) was added. The reaction was stopped by addition of 1 M H_3_PO_4_. Plates were read at 450 nm (reference 570 nm) in a microplate reader (Dynatech). The results were expressed as a percentage of the control level of cytokines production by cells stimulated in the presence of the vehicle of the corresponding compound.

### Determination of serum levels of total and OVA-specific IgE

Serum levels of OVA-specific IgE were measured by IgE ELISA (OptEIA, BD Pharmingen). Briefly, 96-well plates (Maxisorb, Nunc) were coated with OVA (100 μg/ml). After addition of serum samples, a sheep anti-IgE Ab was added to individual wells and its binding was detected with peroxidase-conjugated anti-sheep IgG. IgE concentrations were calculated by comparison with commercial mouse IgE standards (BD Pharmingen).

### Analysis of cytokine mRNA expression by real-time RT-PCR

RNA was prepared from frozen lysates using Rneasy (QIAGEN, Hilden, Germany). One-tube RT-PCR was performed using Quantitect Probe RT-PCR Kit from QIAGEN (Hilden, Germany). Expression of cytokines were determined in relation to beta-actin by real time RT-PCR using TaqMan assay on a ABI Prism 7900. Human primers and probes have been described [6]. Murine primers and probes were Assay-On-Demand purchased from Applied Biosystems (Darmstadt, Germany). Quantity of mRNA was calculated using the ΔΔC_T _method (PE Applied Biosystems User Bulletin #2; ABI PRISM 7700 Sequence Detection System, 1997). For each RT-PCR the threshold cycle (C_T_) was determined, being defined as the cycle at which the fluorescence exceeds 10 times the standard deviation of the mean baseline emission for cycles 3 to 10. Cytokine mRNA levels were normalized to the housekeeping gene β-actin according to the following formula: ΔC_T _= C_T_^β-actin ^- C_T_^cytokine^. Subsequently, respective cytokine mRNA levels were calculated using the ΔΔC_T _method, i.e., ΔC_T _values representing mRNA from cells treated with stimulus in combination with a test compound were set in relation to the ΔC_T _value representing mRNA levels from cells treated with stimulus alone according to the following formula: ΔΔC_T _= ΔC_T_(drug) - ΔC_T_(vehicle). The relative mRNA level for the respective test compound was calculated as 2^-ΔΔC^_T _* 100% based on the results of control experiments with an efficiency of the PCR reaction of approximately 100%.

### Western Blotting analysis of the MAPKs

Human CD4^+ ^T cells were stimulated with Dynabeads CD3/CD28 T Cell Expander (Dynal Biotech GmbH, Hamburg, Germany) or with Dynal beads coated with α-CD3/B7-H2 Fc for 0, 5, 15, 60 or 120 min. After stimulation, cells were washed with ice-cold PBS and lysed in cell lysis buffer (Tris 50 mM, pH 7.2, containing 10 mM EDTA, 150 mM NaCl, 1% NaDOC, 0.1% SDS, 1% Triton X-100) supplemented with protease inhibitors (1 mM PMSF, 5 μg/μl Aprotinin and 5 μg/μl Leupeptin). Cells were left on ice for 20 min and then centrifuged at 14.000 rpm for 10 min. The supernatants were stored at -80°C until measurement of the MAPKs. Equal amounts of protein were resolved in 10% SDS-polyacrylamid gel electrophoresis and transferred onto PVDF membrane. Membranes were blocked overnight at 4°C with 0.2 % I-block (Applied Biosystems, Darmstadt, Germany) in TBS/T buffer and probed with rabbit primary antibodies specific for the dually phosphorylated active forms of ERK, p38 and JNK (Promega GmbH, Mannheim, Germany) for 2 h at room temperature. Blots were washed, incubated 1 h at room temperature with second antibody (donkey anti-rabbit horseradish peroxidase conjugated) (Promega GmbH, Mannheim, Germany). Immunoreactive bands were visualized by enhanced chemiluminiscence (ECL) according to the manufacturer's recommendations (Amersham Pharmacia Biotech, Freiburg, Germany). After detection of the active form of the MAPK, membranes were stripped and reprobed with the corresponding antibody that recognize both active and inactive forms of p38, JNK (Santa Cruz Biotechnology, Inc., Heidelberg, Germany) and ERK (Promega GmbH, Mannheim, Germany).

### ELISA detection of activated JNK

The FACE (Fast Activated Cell-based ELISA) kit (Active Motif, Rixensart, Belgium) was used according to the provider's protocol. CD4^+ ^T cells were cultured in precoated (poly-L-lysine) (Sigma, Deisenhofen, Germany) 96-well plates at a concentration of 1 × 10^6 ^/ml and were stimulated with α-CD3/α-CD28 and α-CD3/B7-H2 Fc for different time points (1 min, 5 min, 15 min, 60 min and 120 min) to induce the JNK pathway. Following stimulation the cells were fixed in order to preserve protein modifications, including phosphorylation. Corresponding wells were then incubated with a primary antibody specific for pJNK and total JNK respectively. Subsequent incubation with secondary HRP-conjugated antibody and developing solution was followed by a colorimetric detection. The signals were then normalized for cell number using Crystal Violet.

### Murine model of late phase eosinophilia

Male Balb/c mice weighing 22–25 g were used. Animals were purchased from Harlan (Borchen, Germany). The animals were kept under constant environmental conditions (temperature: 18 ± 2°C, humidity: 40–60 %, light cycle: 7 am – 7 pm). They had free access to standardized food pellets (purchased from Altromin, Lage, Germany) and tap water. All animal studies were performed in accordance with the national animal protection rules and permitted by the local governmental authority (Regierung von Mittelfranken, Germany). The sensitization strategy was adapted from previously described mouse model [29]. Briefly, all Balb/c mice were actively immunized and boosted by intraperitoneal injections with 200 μl of a solution containing 100 μg ovalbumin (grade V, Sigma, Taufkirchen, Germany) adsorbed on 4 mg of an aqueous solution of aluminium hydroxide and magnesium hydroxide (Alum) (Perbio Science, Bonn, Germany) on days 1, 14 and 20. On day 26, the animals were used for experiments. Compounds, as suspension in 5% DMSO, were given intraperitoneally two hours prior to challenge once a day. Control animals (negative and positive) received only vehicle solution. Mice were challenged twice a day for 4 days by intranasal application of 50 μl of 2 μg/μl ovalbumin solution in NaCl to provoke an influx of inflammatory cells into the airways. Negative control mice were sensitized intraperitoneally with OVA/Alum and were challenged intranasally with saline solution. Positive control animals were sensitized as mentioned above and challenged intranasally with ovalbumin. On day 30 animals were sacrificed by putting them into a CO_2 _rich atmosphere and a bronchoalveolar lavage (BAL) was performed by flushing the lungs and the airways six times with 0.5 ml Hank's balanced solution (Gibco, Karlsruhe, Germany) supplemented with Na-EDTA and HEPES (Sigma, Taufkirchen, Germany). The number of eosinophils as well as the total cell number from the pooled BAL samples of one animal was counted using a haemocytometer (Sysmex microcellcounter F-300, Norderstedt, Germany). BAL cells were spun onto glass slides using a cytospin centrifuge and stained with Diff-Quik (Dade-Behring, Marburg, Germany). The percentage and number of eosinophils were determined microscopically using standard cytological (morphological and staining) criteria by counting 400 cells/slide. Each group of animals treated with compounds was compared with saline challenged (negative control) and vehicle-treated ovalbumin challenged (positive control) groups. For RNA analysis cells recovered from broncheoalveolar lavage were sedimented by centrifugation and RNA was prepared from the cell pellet.

### Histological examination of lungs

Lungs removed from the chest were instilled intratracheally with 0.8 ml 4% buffered formalin solution and immersed in this fixative for 48 hours. Tissues were embedded in paraffin and sections of 5 μm were cut. Slices were stained with May-Grünwald Giemsa for examining inflammation and eosinophilic infiltration and periodic acid-Schiffs stain was done for measuring mucus production under the light microscope. To determine the severity of peribronchial inflammation a semiquantitatively score described by Myou et al. [30] was determined. Briefly, this score describes followingcategories: 0, no inflammation; 1, few inflammatory cells; 2, a ring of inflammatorycells 1 cell layer deep; 3, a ring of inflammatory cells 2–4 cells deep; 4, a ring of inflammatory cells of >4 cells deep. The numerical scores for the abundance of PAS-positive mucus-containingcell in each airway were adapted from the same reference as follows: 0, <0.5%PAS-positive cells; 1, 5–25%; 2, 25–50%; 3, 50–75%;4, >75%.

### Data analysis

Data are expressed as means ± s.e.mean. Significant differences were statistically analyzed by the unpaired Student's *t-*test and by ANOVA. IC_50 _and ED_50 _values were calculated using the computer program PRISM 3.0 (GraphPad Software Inc., San Diego, CA, U.S.A).

## Results

### Influence of coreceptors on TCR induced cytokine expression in CD4^+ ^T cells

**Figure 1:**
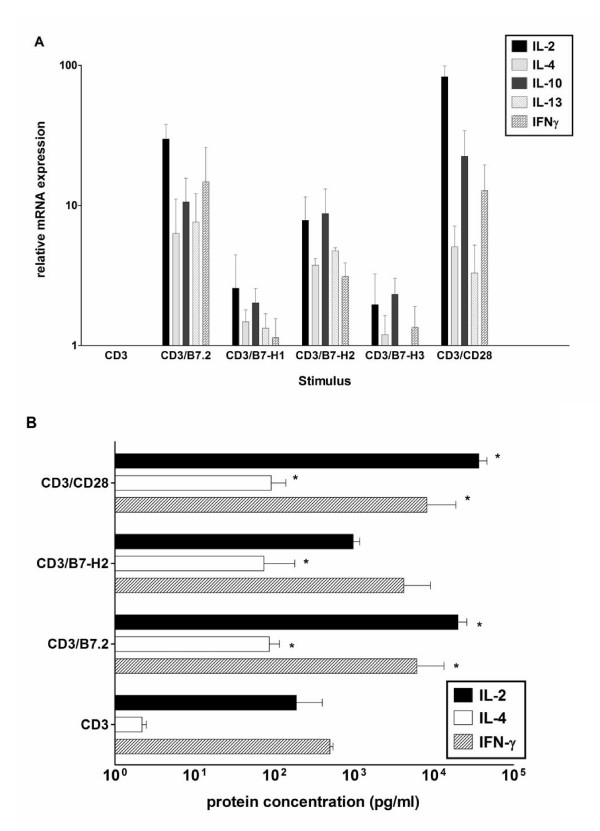
**Induction of cytokines in CD4+ T cells by different costimulatory receptors. **Human CD4^+ ^T cells were stimulated with various stimuli. (A) Cytokine mRNA level were determined after eight hours stimulation using real-time RT-PCR. mRNA level were normalized to β-actin and CD3 stimulated cells were set to 1. Each column represents mean ± s.e.mean of three different donors. (B) Cytokine protein level was determined in the supernatant by ELISA after twenty-four hours of stimulation. Each column represents mean ± s.e.mean of three different volunteers. *P < 0.05 (versus CD3 stimulated samples).

The stimulation of human CD4^+ ^T cells by beads coated with α-CD3 antibodies and various ligands for coreceptors was compared. For our studies, we developed a real-time RT-PCR method for accurate quantization of T cell cytokines. Cells were stimulated with beads coated with α-CD3 alone, α-CD3 plus α-CD28, α-CD3 plus B7-2 Fc fusion protein, α-CD3 plus B7-H2 Fc fusion protein, α-CD3 plus PD-1 ligand Fc fusion protein (PD-L1 or B7-H1) or α-CD3 plus B7-H3 Fc fusion protein. Naked beads alone were included as a negative control and did not activate CD4^+ ^T cells. IL-5 mRNA was not induced by any stimulation condition. In contrast, IL-2, IL-4, IL-10, IL-13 and IFNγ mRNA expression was already stimulated by α-CD3 activation alone and maximally stimulated by α-CD3/α-CD28 activation, which was included as a positive control (Figure 1A). B7-2 and B7-H2 binding to their respective counterreceptors costimulated cytokine gene expression to elevated levels compared to α-CD3 activation alone. Costimulation strength by ICOS ligation was comparable to CD28 costimulation. In contrast, B7-H1 and B7-H3 mediated costimulation did not augment α-CD3 induced cytokine mRNA levels (Figure 1A). To substantiate these results, cytokine protein concentration of the prototypic cytokines IL-2, IL-4 and IFN was determined in stimulated CD4^+ ^T cell supernatants (Figure 1B). The pattern was similar to the induction on the mRNA level. B7-2 and B7-H2 costimulation produced more cytokines than α-CD3 stimulation alone. B7-2 was more effective in costimulation that B7-H2 (Figure 1B). Similarly, B7-H1 and B7-H3 mediated costimulation did not augment α-CD3 induced cytokine protein levels (data not shown).

### Time course of cytokine induction in CD4^+ ^T cells

**Figure 2:**
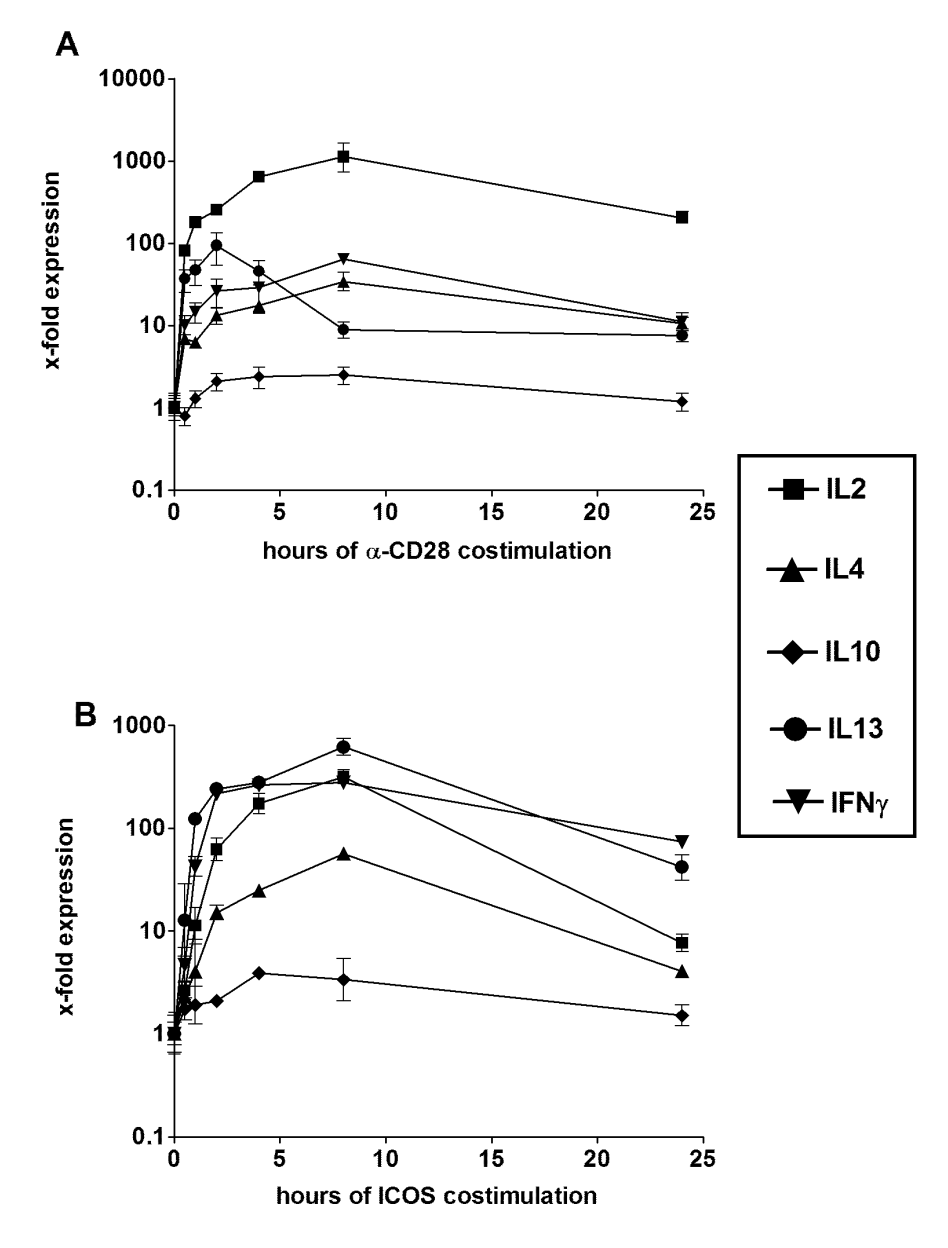
**Time course of cytokine induction by costimulatory receptors. **Human CD4^+ ^T cells were stimulated by α-CD3/B7-2 (A) and α-CD3/B7-H2 (B) respectively and harvested after different time points. Cytokine mRNA level were determined using real-time RT-PCR. Levels were normalized to β-actin and unstimulated cells were set to 1. Each point and bar represents mean ± s.e.mean of duplicates measurements of one donor. Similar results were obtained from three independent donors.

Since CD28 and ICOS were the only stimulating coreceptors, they were chosen to study the time course of cytokine mRNA induction. As can be seen from figure 2A, α-CD3/B7-2 stimulation induced cytokine mRNA levels rapidly till two hours. Whereas IL-13 mRNA levels started to decline from that time point on, other cytokine mRNA levels still increased. Similar observations were made after α-CD3/B7-H2 stimulation (Figure 2B). Most remarkable, IL-13 and IFNγ were more strongly induced as compared to α-CD3/B7-2 stimulation, whereas IL-2 was much weaker induced. The induction of IL-10 was weak for both costimulatory receptors.

### Activation of MAPKs by costimulatory receptors

**Figure 3:**
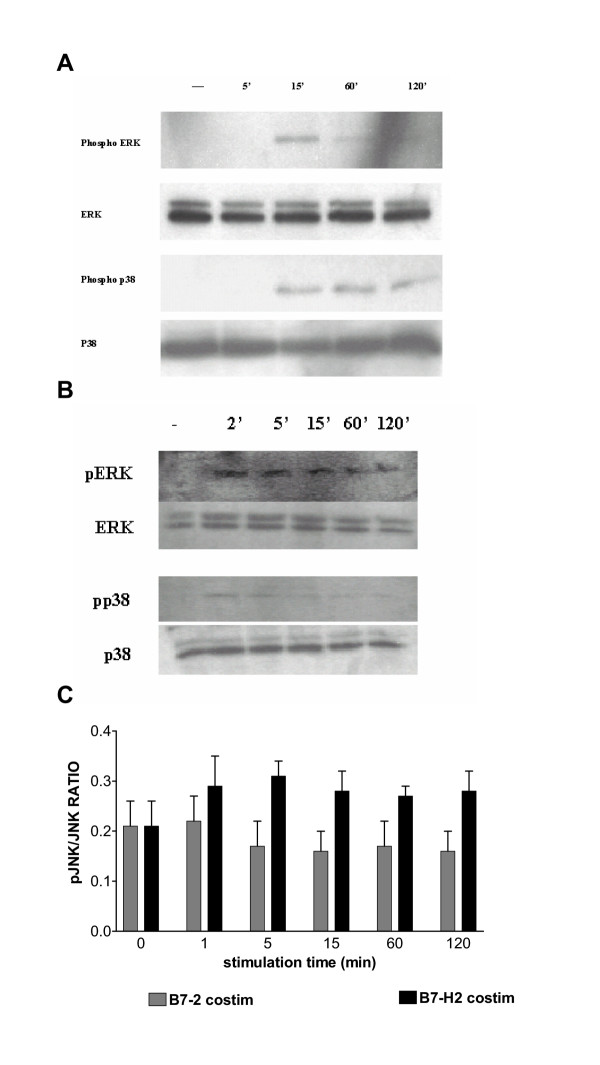
**Activation of MAPKs in human primary CD4+ T-cells by costimulatory receptors. **Human CD4^+ ^T cells were seeded at a concentration of 1 × 10^6^/ml and stimulated with Dynal beads coated with α-CD3/B7-2 Fc (A) or α-CD3/B7-H2 Fc (B) for 0, 2, 5, 15, 60 or 120 min as indicated. Cells were harvested and cell extracts prepared. Cell extracts were analyzed by Western blot using either Anti-active pAb (upper row) or with corresponding antibodies that recognize both active and inactive forms of each subfamily of MAPK. Results are representative of three experiments with similar results. (C) Activated JNK was detected by ELISA. CD4^+ ^T cells were cultured in precoated 96-well plates at a concentration of 1 × 10^6 ^/ml. Cells were stimulated with α-CD3/B7-2 Fc and α-CD3/B7-H2 Fc for different times (1, 5, 15, 60 and 120 min). Following stimulation the cells were fixed. The phosphorylated form of JNK as well as the amount of total JNK was detected using an ELISA method. Results shown are representative for three independent experiments.

We speculated that differential activation of MAPK family members by CD28 and ICOS may contribute to their different cytokine production patterns. To this end, we investigated the activation of the MAPK family members as downstream signaling molecules. T cell receptor and B7-2 (Figure 3A) or B7-H2 (Figure 3B) cross-linking elicited the phosphorylation of ERK and p38 indicating the up-regulation of kinase activity. The activation reached a maximum 15 min after B7-2 costimulation and 2 min after B7-H2 costimulation for both kinases. It persisted over a period of two hours. Since we could not detect JNK by western blot, we analyzed JNK activation by a recently available ELISA method. A cell-based ELISA method was used to determine the JNK phosphorylation relative to the total JNK protein found in CD4^+ ^T cells. Stimulation with α-CD3/α-CD28 and α-CD3/B7-H2 for different time points was assessed to induce the JNK pathway. No effect could be observed in cell costimulated with α-CD3/α-CD28. In CD4^+ ^T cells costimulated with α-CD3/B7-H2 an increase in the ratio of phosphorylated JNK to total JNK was observed (Figure 3C).

### Effect of MAPK inhibitors on costimulated CD4^+ ^T cells

These results indicated a role of MAPKs in the signaling cascade precipitated from the costimulatory receptors CD28 and ICOS leading to cytokine expression. To elucidate which MAPK is important for cytokine induction, we first analyzed whether selective MAPK inhibitors were able to inhibit α-CD3/B7-2 induced gene expression. To this end, CD4^+ ^T cells were preincubated with three selective MAPK inhibitors: SP600125 (selective inhibitor of c-Jun N-terminal kinase (JNK)), U0126 (selective inhibitor of MEK-1 and MEK2) and SB203580 (selective inhibitor of p38 MAPK). These compounds were chosen based on their pharmacological profile (high potency and specificity) for these kinases [31-33]. Since the cytokine induction behaved similar on the protein and mRNA level (see above), only mRNA was taken into consideration for determination of IC_50 _values for inhibition of cytokines. The JNK inhibitor did not affect induction of any cytokine analyzed. The p38 inhibitor inhibited IL-2, IL-13 and IFNγ with a similar IC_50 _of 3–7 μM, whereas IL-4 gene expression was not affected (Table 1). The MEK inhibitor U0126 inhibited IL-2 and IFNγ gene expression with IC_50 _of about 1 μM. IL-4 gene expression was not affected, whereas IL-13 was potently inhibited with an IC_50 _of 0.18 μM (Table 1). Since ICOS costimulation also induced the secretion of various cytokines and activated MAPKs, we further analyzed whether this induction is influenced by different pharmacological inhibitors of MAPKs in the same manner as for α-CD3/B7-2 activation. The selective p38 MAPK inhibitor (SB203580) affected cytokine synthesis only at very high concentrations (>30 μM). The JNK inhibitor SP600125 inhibited IL-2, IL-13 and IFNγ dose dependently at similar concentrations of about 1 μM whereas IL-4 was not inhibited by this compound (Table 1). The ERK inhibitor U0126 inhibited IL-2, IL-13 and IFNγ dose dependently at similar concentrations of 0.1 to 0.4 μM whereas IL-4 was inhibited only at ten-fold higher doses.

**Table 1:** IC_50 _values for the inhibition of cytokine mRNA synthesis by different MAPK inhibitors in CD28 or ICOS costimulated human CD4+ T cells. Human CD4+ T cells were preincubated with increasing concentrations of SP600125, U0126 and SB203580. The cells were then stimulated with beads coated with α-CD3/B7-2 fusion protein or α-CD3/B7-H2 fusion protein for 8 hours. Cytokine mRNA level were determined in triplicates using real time RT-PCR and were normalized to β-actin. Stimulated cells treated with DMSO were used as 100% control. IC_50_values were calculated for the different cytokines. Results represent mean ± s.e.m. for three different donors.

	*α-CD3/B7-2 stimulation*			
**μM**	**IL-2**	**IL-4**	**IL-13**	**IFNγ**

**U 0126**	1.17 ± 0.97	no effect*	0.18 ± 0.14	0.72 ± 0.39
**SB 203580**	6.99 ± 4.57	no effect*	3.02 ± 1.14	4.15 ± 1.38
**SP 600125**	no effect*	no effect*	no effect*	no effect*
	*α-CD3/B7-H2 stimulation*			

**μM**	**IL2**	**IL4**	**IL13**	**IFNγ**

**U 0126**	0.11 ± 0.03	1.29 ± 0.96	0.31 ± 0.09	0.14 ± 0.08
**SB 203580**	no effect*	no effect*	no effect*	6.93 ± 2.47
**SP 600125**	1.25 ± 0.18	no effect*	1.33 ± 0.36	0.79 ± 0.35

*concentrations up to 30 μM were tested

### Effects of MAPK inhibitors *in vivo*

**Figure 4:**
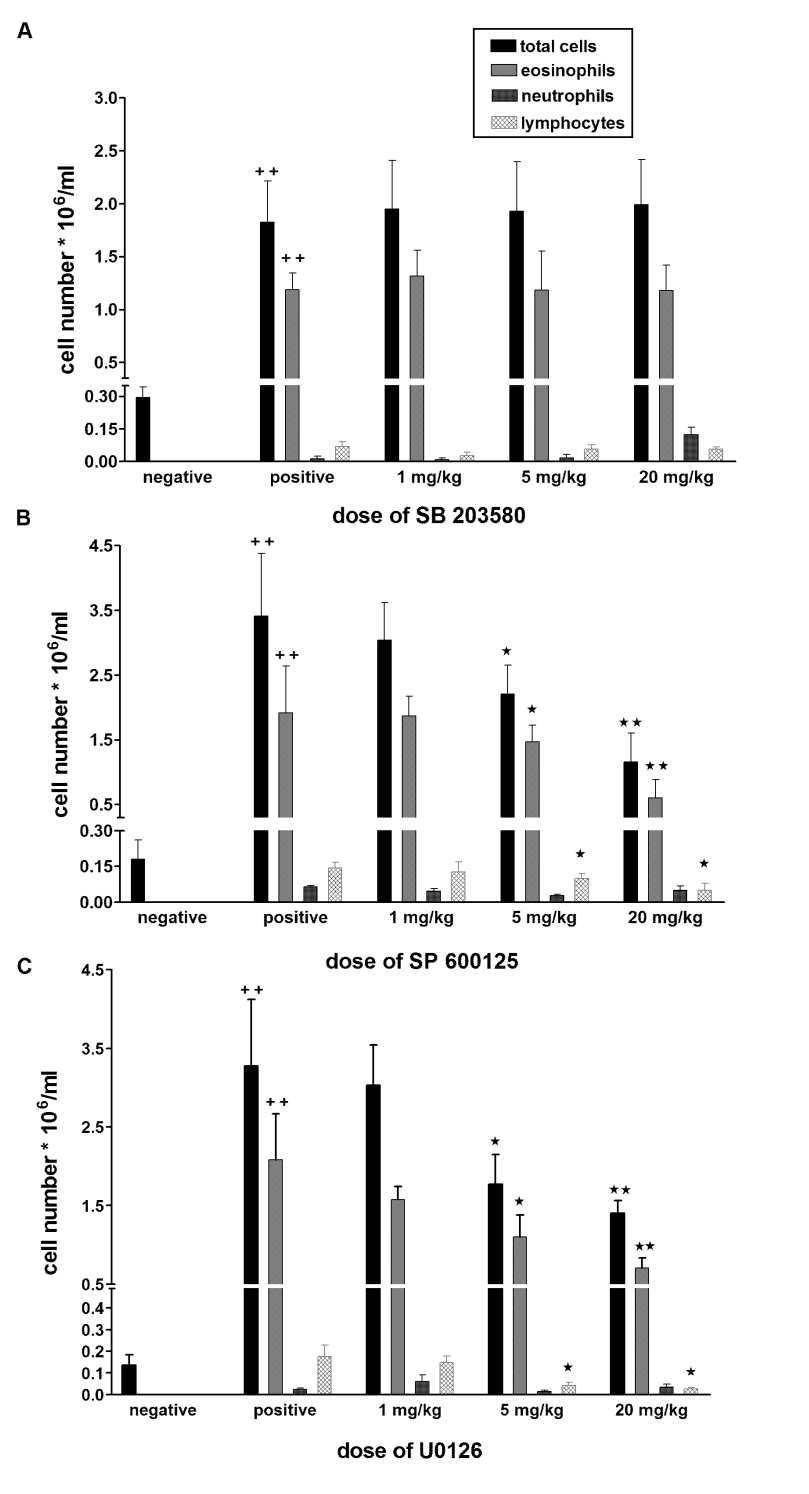
**Effects of MAPK inhibitors *in vivo***. Actively intraperitoneally sensitized mice were challenged by intranasally administration of ovalbumin. SB203580 (Figure A), SP600125 (Figure B) and U0126 (Figure C) were given intraperitoneally two hours prior to ovalbumin challenge. After 4 days of challenge bronchoalveolar lavage was performed. Total cell number in BAL and differential cell count on cytospin preparations was assessed. OVA challenged vehicle-treated animals were taken as a positive control. Each column represents mean ± s.e.mean. n = 5 mice per group. *p < 0.05 (drug treated versus OVA challenged vehicle (positive) control), **p < 0.005 (drug treated versus OVA challenged vehicle (positive) control). ^++ ^p < 0.005 (OVA challenged (positive) versus saline challenged (negative) control).

**Figure 5:**
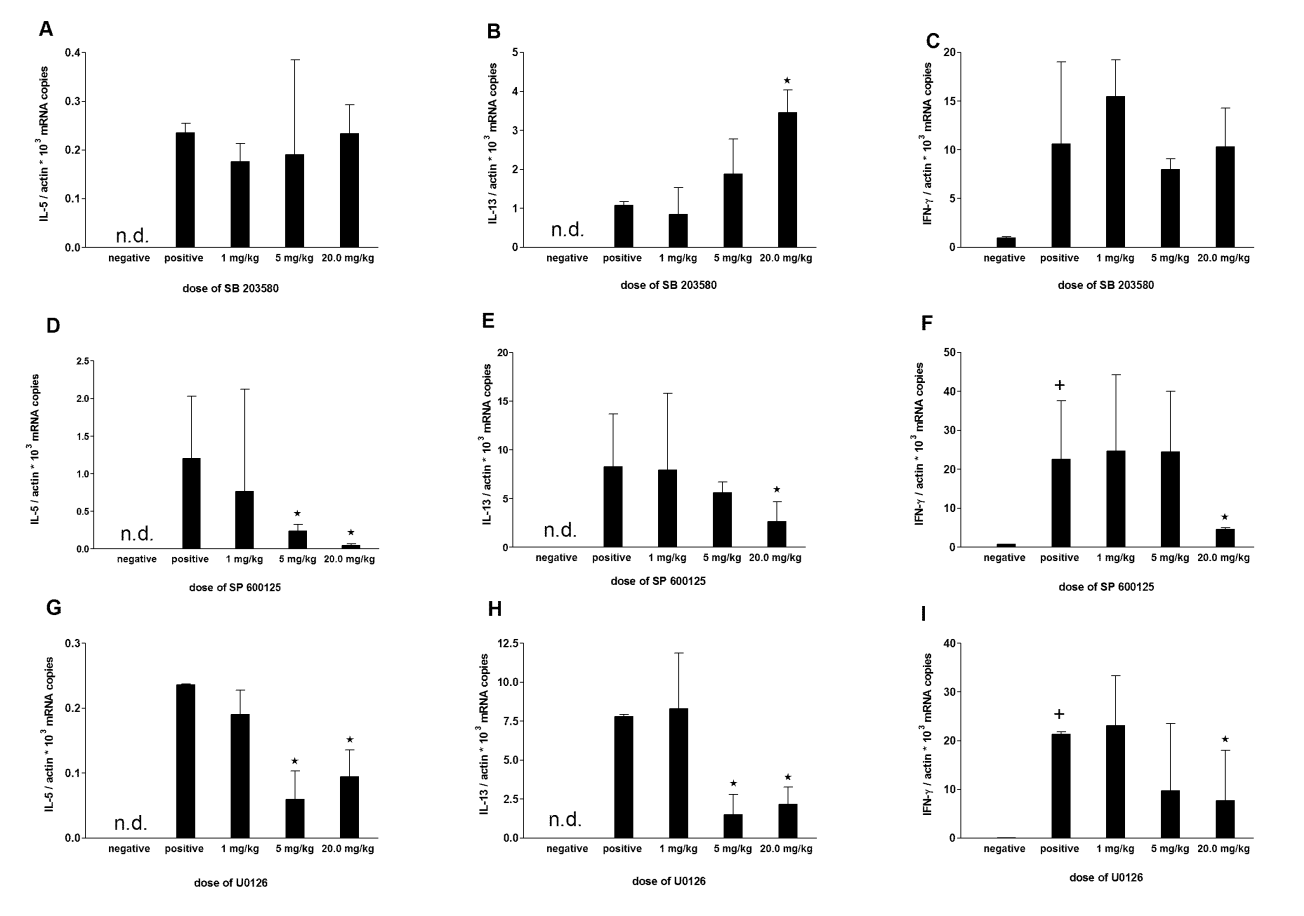
**Effects of MAPK inhibitors on BAL cytokine levels. **Cells were recovered from bronchoalveolar lavage by centrifugation and RNA was prepared from the cell pellet. Cytokine mRNA level were determined using real-time RT-PCR and were normalized to β-actin. Each column represents mean ± s.e.mean. n = 5 mice per group. n.d. – not detectable. *p < 0.05 (versus OVA challenged positive control), ^+^p < 0.05 (negative versus positive control)

The finding that the MAPKs inhibitors very effectively inhibited several cytokines in CD4^+ ^T cells costimulated through CD28 and ICOS, prompted us to investigate whether these selective MAPKs inhibitors may be active in an animal model of allergic asthma. BALB/c mice were actively sensitized to ovalbumin and challenged by intranasal application of ovalbumin solution. Different doses of the three selective MAPKs inhibitors SP600125, U0126, SB203580 were given intraperitoneally 2 hours before challenge. After 4 days of challenge mice were killed and bronchoalveolar lavage (BAL) was performed. Antigen challenge caused a strong eosinophilic inflammation of the lung and this was reflected in the cellular composition of the lung lavage fluid. Compared with saline challenged animals, the allergen challenge in sensitized animals led to an increase in all leukocyte populations especially of eosinophils in the lavage fluid (Figure 4). On average, the total amount of leucocytes in the BAL of the positive control animals was 2.26 × 10^6 ^cells /ml (n = 7) and eosinophils accounted for 60.16 % of the total cell count, whereas in the saline challenged animals 0.16 × 10^6 ^cells /ml (n = 7) could be detected, which contained no granulocytes or lymphocytes. The treatment of the animals with SB203580 hardly affected leukocyte and eosinophils numbers in the lavage fluid, even at very high doses (20 mg/kg) (Figure 4A). In contrast, the administration of U0126 and SP600125 inhibited recruitment of the total cell number in the bronchoalveolar lavage dose dependently with an ED_50 _value of 2.5 mg/kg and 9 mg/kg respectively. Absolute eosinophil and lymphocyte numbers were also dose dependently inhibited by the ERK and JNK inhibitors, whereas neutrophil numbers were not affected. There were minor but not significant relative changes in the different cell populations. The cytokine protein levels in BAL fluid were below the detection limit of the ELISA kits used. Therefore cytokine mRNA levels were determined from BAL cells. SB203580 lacked also any effect on the cytokine mRNA induced in BAL cells (Figure 5A–C). In contrast, SP600125 and U0126 dose dependently inhibited IL-5 mRNA induced in BAL cells (Fig. 5D, G). Both compounds inhibited also IL-13 and IFNγmRNA at higher doses (Fig. 5 E, F, H, I).

### Effects of MAPK inhibitors on serum OVA-specific IgE levels

**Figure 6:**
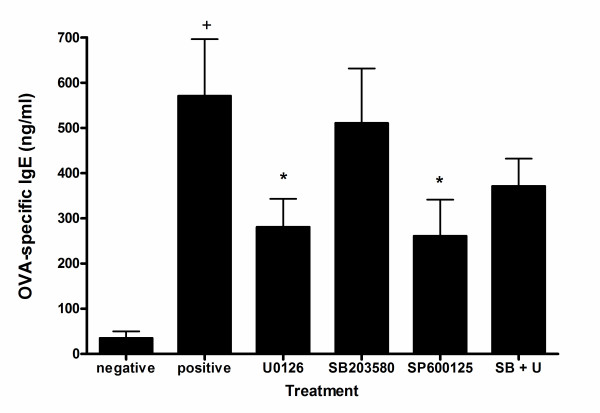
**Effects of MAPK inhibitors on OVA-specific IgE antibody levels in the serum. **Mouse serum was collected 24 h after the last OVA challenge. Samples were obtained from saline- (negative) or OVA-challenged mice in the absence (positive) or presence of MAPK inhibitors: SB203580 (20 mg/kg), U0126 (20 mg/kg), SP600125 (20 mg/kg) or of the combination of U0126 (5 mg/kg) plus SB203580 (5 mg/kg). Serum titers for OVA-specific IgE antibodies were measured by ELISA as described in methods. Each column represents mean ± s.e.mean. n = 5 mice per group. *p < 0.05 (versus OVA challenged positive control), ^+^p < 0.05 (negative versus positive control)

We next determined whether MAPK inhibitors could modify an ongoing OVA-specificTh2 response *in vivo *by analyzing OVA-specific IgE levels. Serum was collected24 h after the last OVA challenge. Levels of OVA-specfic IgE, was determined using ELISA. Substantial elevation OVA-specific IgE was observed in serum from OVA-sensitized and -challengedmice as compared with untreated mice (Fig. 6). U0126 and SP600125 significantly lowered total OVA-specific IgE levels, whereas SB203580 did not affect OVA-specific IgE levels.

### Effects of MAPK inhibitors on lung inflammation *in vivo*

**Figure 7:**
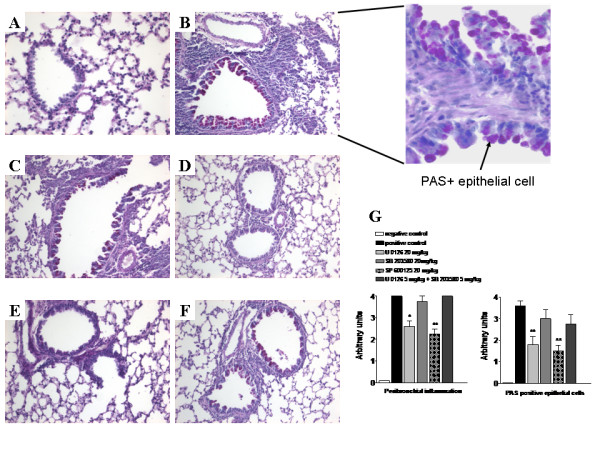
**Histology of lung inflammation. **Representative sections of the lungs are shown. Sections are periodic acid-Schiff stained and counterstained with haematoxylin for analysis of mucin-containing cells. Tissue was examined by light microscopy (original magnification 20×), zoomed inset delineates PAS+ epithelial cells (100×). Lung sections were obtained from saline- (A) or OVA- (B, C, D, E, F) challenged mice in the absence (A, B) or presence of MAPK inhibitors: SB 203580 (20 mg/kg) (C), U 0126 (20 mg/kg) (D), SP 600125 (20 mg/kg) (E) or of the combination of U 0126 (5 mg/kg) plus SB 203580 (5 mg/kg) (F). Peribronchial inflammation and the abundance of PAS-positive mucus-containing cells were determined by applying the scores as described in methods (G). The scores described in methods were applied to ten bronchi for each animal and mean and standard deviation were determined for all the animals in a group (n = 5). *P < 0.05 and **P < 0.01 compared with positive control group.

On separate experiments, lung tissue was collected 24 hours after the last challenge. Ovalbumin challenge induced marked infiltration of inflammatory cells into the peribronchial and perivasular tissue as compared with saline challenge (Figure 7 A, B). The majority of the infiltrated inflammatory cells were eosinophils as detected in the May-Günwald Giemsa stain (data not shown). MAPK inhibitors had different effects on peribronchial inflammation. The inflammatory score (calculated as described in Methods) decreased significantly after administration of 20 mg/kg ERK or JNK inhibitor, whereas inhibition of p38 had no influence on peribronchial inflammation (Figure 7G). The allergen challenge induced mucus secretion was detected with periodic acid-Schiff stain counterstained with hematoxylin (Figure 7A, B). The percentage of PAS-positive mucus-containing epithelial cells decreased as an effect of ERK or JNK inhibition (Figure 7D, E, G). The p38 inhibitor had no effect on mucus production (Figure 7C, G).

### Effects of combined administration of MAPK inhibitors

**Figure 8:**
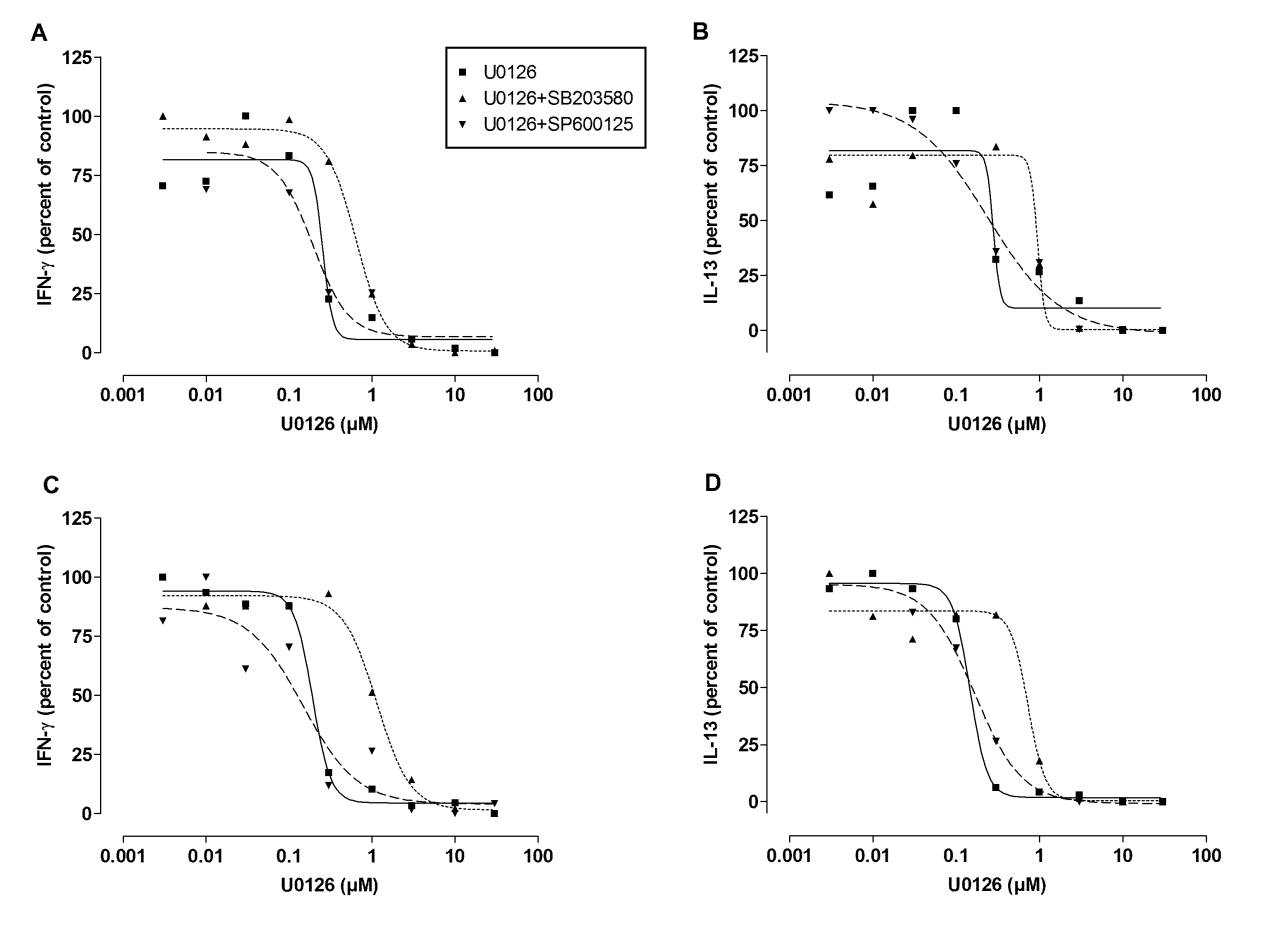
**Effects of combined administration of MAPK inhibitors *in vitro***. Human CD4^+ ^T cells were preincubated either with increasing doses of U0126 alone (solid line) or in combination with SP 600125 (dashed line) of with SB 203580 (dotted line). Doses of the MAPK inhibitors are specitied in the text. After that, cells were stimulated with α-CD3/B7-2 Fc (A, B) or α-CD3/B7-H2 Fc (C, D) for 8 hours. IFNγ (A, C) and IL-13 (B, D) cytokine mRNA was measured by real-time RT-PCR. Dose-response curves were determined and IC_50 _values were calculated. Data are representative of three different donors.

**Figure 9:**
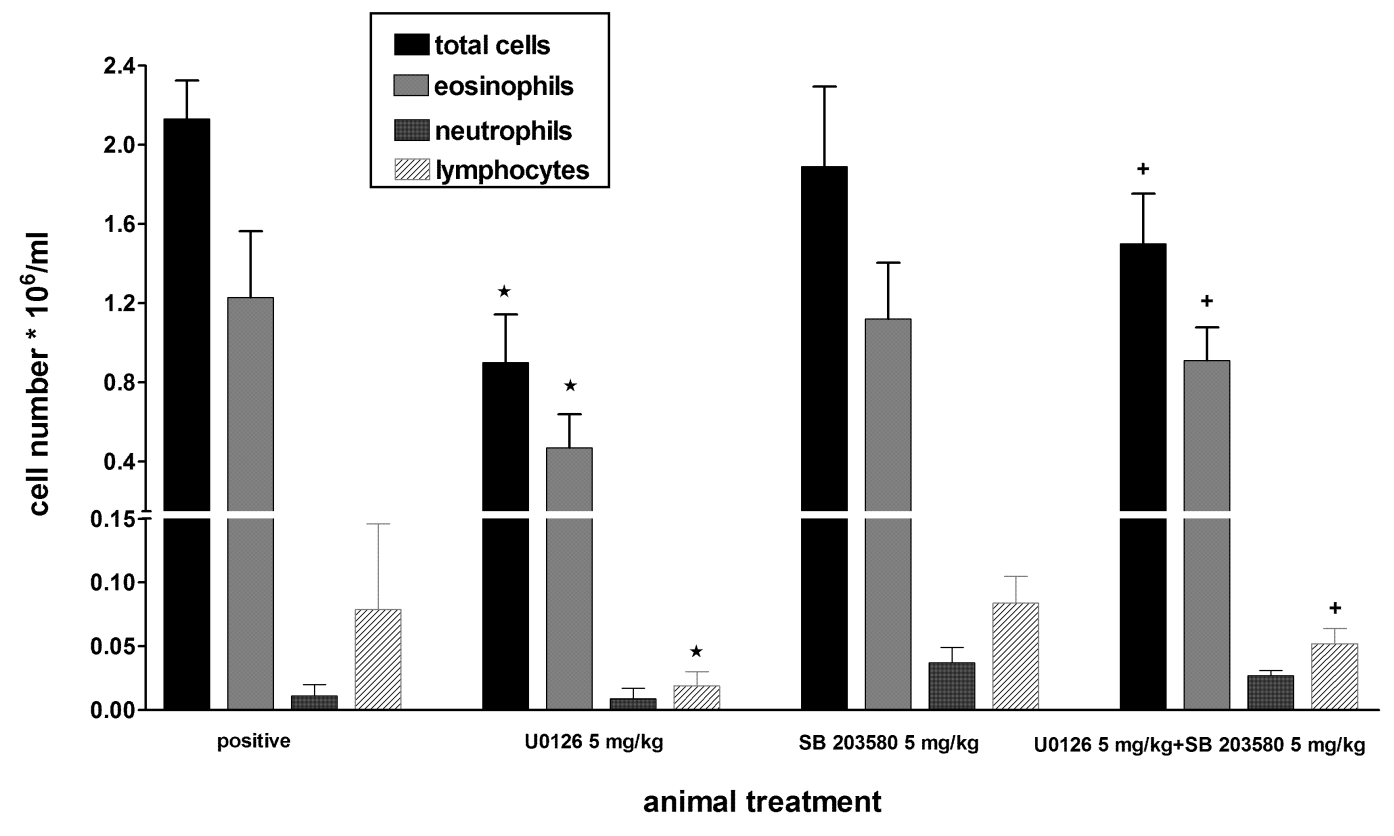
**Effects of combined administration of MAPK inhibitors *in vivo***. Late phase eosinophilia was induced in mice pretreated either with 5 mg/kg U0126 and 5 mg/kg SB 203580 or with the combination thereof. BAL was performed and total cell number and differential leukocyte count was determined. OVA challenged vehicle-treated animals were taken as positive control. Each column represents mean ± s.e.mean. n = 5 mice per group. *p < 0.05 (versus OVA challenged vehicle control), ^+^p < 0.05 (versus U0126 alone).

**Figure 10:**
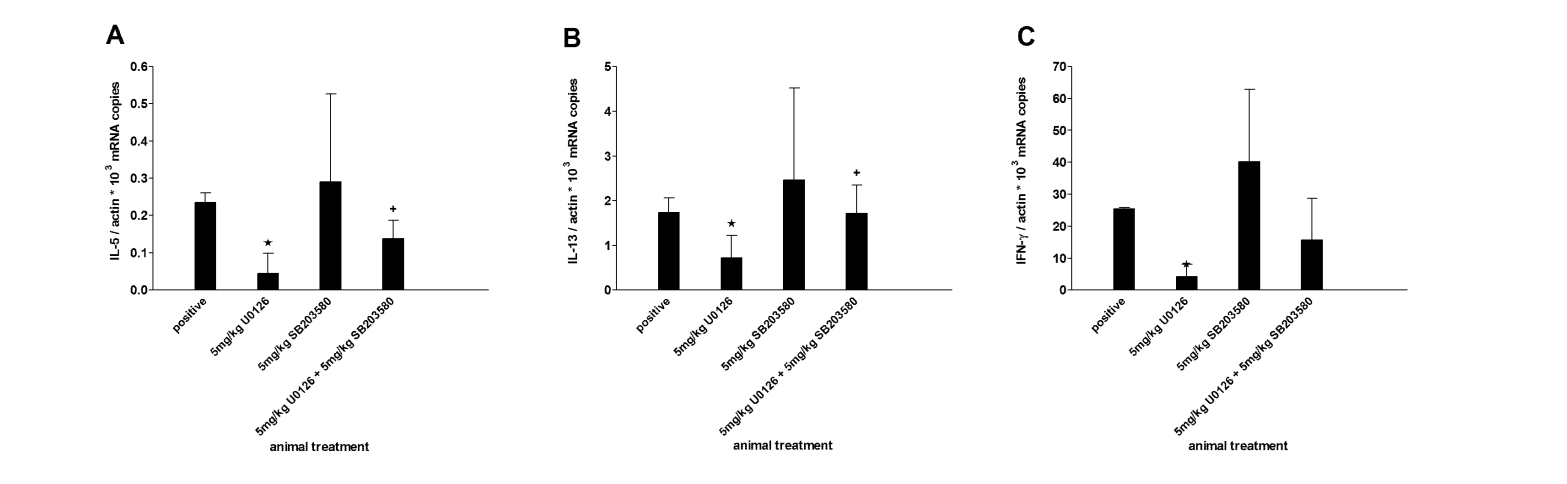
**Effects of combined MAPK inhibitors on BAL cytokine levels. **Cells were recovered from bronchoalveolar lavage by centrifugation and RNA was prepared from the cell pellet. Cytokine mRNA level were determined using real-time RT-PCR and were normalized to β-actin. Each column represents mean ± s.e.mean. n = 5 mice per group. *p < 0.05 (versus OVA challenged vehicle control), ^+^p < 0.05 (versus U0126 alone).

Since the three MAPKs are part of different signaling cascades with different upstream and downstream mediators, we asked whether the combination of two MAPK inhibitors may have synergistic effects on the induction of cytokines in CD4+T cells. The simultaneous addition of SB203580 (5 μM) and SP600125 (3 μM) did not cause any synergistic effect. Unexpectedly, the simultaneous administration of increasing concentrations of U0126 (0.01 μM to 30 μM) and SB 203580 to α-CD3/α-B7-2 (Figure 8A, B) and α-CD3/B7-H2 (Figure 8C and 8D) stimulated CD4^+ ^T cells led to a shift to the right of the dose-response curve for U0126 compared to the administration of U 0126 alone. The dose for SB203580 was 10 μM for ICOS costimulation and 1 μM for CD28 costimulation, respectively. This was reflected by an increase in the corresponding IC_50 _values (Table 2). In contrast, the addition of SP600125 (0.3 μM for ICOS costimulation and 1.5 μM for CD28 costimulation) did not change the dose response curve for U0126 (Figure 8). Since we observed for the combination SB203560 and U0126 only unforeseen effects *in vitro*, we analyzed this combination *in vivo*. The simultaneous treatment of the animals with 5 mg/kg SB203580 and 5 mg/kg U0126 (Figure 9) diminished the inhibition of late phase eosinophilia by the solely administration of the ERK inhibitor. There were minor and not significant change in the relative numbers of the populations counted in the BAL. This effect could also be observed measuring cytokine mRNA levels in the BAL cells. Treatment of the animals with SB203580 antagonized the inhibiting effect of U0126 on IL-5, IL-13 and IFNγ mRNA level (Figure 10). The effect of the combined administration of ERK inhibitor and p38 inhibitor on lung histology had the same effect as observed *in vitro*, as well as on the cellular composition of the BAL. The p38 inhibitor abolished the effect of ERK inhibition when administered simultaneously compared to administration of ERK inhibitor alone (Figure 7F, G). Furthermore, OVA-specific IgE Level in the serum of mice treated with the combination of the ERK and p38 inhibitor were higher than in mice treated with the ERK inhibitor alone (Figure 6).

**Table 2:** IC_50 _values for the inhibition of cytokine mRNA synthesis by combined administration of MAPK inhibitors *in vitro*. IC_50_values were calculated from data shown in Figure 8.

**μM**	**IFNγ (U0126)**	**IFNγ (U0126+SB203580)**	**IL-13 (U0126)**	**IL-13 (U0126+SB203580)**
α-CD3/B7-2	0.29	0.68	0.28	0.97
α-CD3/B7-H2	0.19	1.14	0.21	0.73

## Discussion

Costimulation is essential for the full activation of CD4^+ ^T cells. In this study, we analyzed the induction and regulation of Th_1 _and Th_2 _cytokines in CD4^+ ^T cells upon engagement of different costimulatory receptors. Beads with defined combinations of surface receptor stimulating antibodies and costimulatory receptor ligands were applied to induce cytokine synthesis. Ligation of two costimulatory receptors, CD28 and ICOS, augmented TCR activation and this in part is due to MAPK activation. The effects of pharmacological inhibitors of three different MAPKs on cytokines induced by α-CD3/B7-2 or α-CD3/B7-2 were investigated. Using these compounds, we were able to elucidate different MAPK signaling pathways leading to cytokines synthesis in dependence on the costimulatory receptor engaged. We tested these compounds in an animal model of asthma in which the ERK inhibitor U0126 and the JNK inhibitor SP600125 were able to reduce the influx of eosinophils into the lungs of sensitized and challenged mice. We found no synergistic effect of any combination of these MAPK inhibitors. Unexpectedly, SB203580 antagonized the *in vitro *and *in vivo *action of U0126.

For optimal activation, CD4^+ ^T cells require specific antigen recognition by the TCR and additional signals (collectively called costimulatory signals), delivered by the same antigen-presenting cell [34]. In the absence of costimulation, lymphocytes fail to respond effectively and are rendered anergic [35]. CD28 is the best-characterized costimulatory receptor and constitutively present on the surface of T cells (reviewed in [12]). CD28 is activated by B7-1 and B7-2 counter-receptors on antigen-presenting cells. Its signaling contributes to the overall strength of T cell activation [36]. However, new B7- and CD28-like molecules have recently been discovered and new pathways have been delineated that seem to be important for regulating the responses of previously activated T cells [37]. Some B7 homologues have unknown receptors, indicating that other immunoregulatory pathways remain to be described. We established an *in vitro *test system to analyze to role of each coreceptor. To this end, we conjugated beads with defined combinations of α-CD3 and B7 counterreceptors. In line with previous reports described above we found that CD28 ligation augments the synthesis of all T cell derived cytokines. Similar results were obtained by ligating ICOS via its counter receptor ICOS-L (B7-H2). The effects of ICOS signals on T cell proliferation and IL-2 production were reported to be modest in comparison with those of CD28 [16]. However, ICOS costimulation is equivalent to that mediated by CD28 costimulation for the production of effector cytokines like IFNγ, IL-4, IL-10 and IL-13. IL-5 was not detected. It has been shown, that efficient *in vitro *IL-5 production needs elevated cAMP level in addition to TCR stimulation [38]. There was no difference in the strength of induction of Th2 compared with Th1 cytokines for CD28 and ICOS costimulation. Conflicting data has been reported about the function of PD-1. The engagement of PD-1 by its specific ligands, B7-H1 (PD-L1) or B7-DC (PD-L2) was reported to inhibit T and B cell proliferation and cytokine production [39,40]. In our test system engagement of PD-1 by ligation with B7-H1 neither augmented nor inhibited cytokine synthesis.

Activation of MAPK family members by CD28 and ICOS may contribute to their costimulatory activity. To analyze whether different signaling cascades are precipitated by the different coreceptors, their ability to activate different MAPK pathways was determined. Since only CD28 and ICOS activation augmented cytokine synthesis we focused our analyses on these receptors. We analyzed the signaling pathways of ERK1/2, JNK, and p38 MAP kinase, during the primary activation of human T-cells, costimulated with ICOS or CD28. ERK1/2 and p38 kinase were markedly activated by both CD28- and ICOS-mediated costimulation. In our cellular assay system the activation of ERK has a role in T-cell activation via CD28 and ICOS. The activation of ERK by CD28 is mediated by binding of SOS to the YMNM motif of the intracellular domain of CD28 [41]. The YMNM motif is not conserved in ICOS, the sequence being YMFM, and amino-acid substitutions in this motif results in a failure to associate with Grb2 [16,42]. Therefore it is currently unclear, how ICOS is able to activate ERK.

Previous studies of p38 MAP kinase in human purified T cells [43] and in the CD4^+ ^subset [7] clearly demonstrated the involvement of p38 MAP kinase in the cell activation through TCR and CD28 costimulation signal pathways. However, little is known about this MAP kinase in ICOS costimulated T cells. In our cellular assay activation of p38 MAPK by ICOS was detected. This is in line with recent reports, in which ICOS ligation synergized with TCR signals for activation of the ERK and p38 MAP kinases [44,45].

While enhanced activityof JNK is an absolute necessity for regulation of IL-2 geneexpression in T cell lines [46], a defect in JNK signalingwas claimed to be involved in T cell differentiation, but notin T cell activation *in vivo*, i.e. in JNK-deficient animals [47]. This corresponds to our results with human T cells, where JNK was found not to be activated after CD28 costimulation. Conflicting reports appeared about the ability of ICOS to activate the JNK pathway. Parry et al. reported that only CD28 but not ICOS costimulation activated c-jun N-terminal kinase [48]. Arimura et al. reported that the cross-linking of ICOS induced much less phosphorylation of JNK than did the cross-linking of CD28 [49]. In our cellular assay system we found that ICOS activated the JNK pathway. However, this was only detected by using a sensitive ELISA based assay indicating a very low expression of JNK in primary human T cells. Alltogether, whereas p38 and ERK are consistently found to be activated after CD28 and ICOS costimulation, the activation of JNK appeared only after ICOS costimulation.

In order to elucidate which of the activated MAP kinases are important for cytokine induction after T cell activation and costimulation we employed specific inhibitors of the different MAPKs in our cellular assay system. These compounds were chosen based on their pharmacological profile (high potency and specificity) for these three kinases [31-33]. The p38 inhibitor SB203580 and the ERK inhibitor U0126 inhibited IL-2, IL-13 and IFNγ in CD28 costimulated T-cells. IL-4 induction, in contrast, was not affected by these inhibitors. In line with our observations, that JNK does not become activated by CD28 costimulation, the JNK inhibitor did not affect cytokine induction in CD28 costimulated T cells. Only one report analyzed the effect of a p38 inhibitor in primary human CD4^+ ^T cells. There SB203580 blocked human CD4 T cell production of IL-4, IL-5, TNF-, and IFN-, but not IL-2, in response to CD3 and CD28 stimulation [7]. This difference is most likely due to different experimental protocols and the use of a different T cell population. While U0126 was never tested to inhibit human T cell cytokine production, in the first description of the JNK inhibitor SP600125, its effect on polarized Th1 and Th2 cells was described. There Th1 cytokines and some Th2 cytokines but not IL-4 was inhibited by this compound [32]. It is tempting to speculate, that the polarization of the T cells rendered them susceptible to JNK inhibition. Different observations were made in ICOS costimulated T cells. For human T cells, it has only been reported, that U0126 blocked the proliferation of T-cells and the transcription of IL-2 following costimulation by ICOS in human T cell [45]. We found, that the p38 inhibitor was not effective, but the JNK inhibitor inhibited IL-2, IL-13 and IFNγ. The ERK inhibitor U0126 inhibited all cytokines with similar efficiency as in CD28 costimulated T cells. Together these data indicates that in human T cells ICOS precipitates MAPK signaling cascades differentially compared to murine T cells. More interestingly, our inhibitor studies revealed that not every MAPK signaling cascade activated by costimulation is necessary for cytokine synthesis.

The sensitivity of CD4^+ ^T cells to various MAPK inhibitors prompted us to analyze these compounds in an animal model of asthma. The p38 inhibitor SB203580 did not affect airway inflammation in our model. Conflicting data about the action of p38 inhibitors in asthma models have been reported in the literature. Similar to our results, one study found no effect of SB203580 in rats [50]. In contrast, Underwood et al. demonstrated anti-allergic activity using a novel second-generation p38 MAPK inhibitor [51]. Either increased specificity of this new inhibitor or better bioavailability of this compound may explain its effect *in vivo*. In contrast to the p38 inhibitor, the ERK as well as the JNK inhibitor dose dependently decreased allergen induced airway eosinophilia in our model. This anti-inflammatory effect of the ERK and the JNK inhibitor was also detected by measuring peribronchial inflammation and mucus production. Elevated serum IgE is a hallmark of a Th2 immune response. Our data showed that serum levels of OVA-specific IgE were also substantially reduced by U0126 and SP600125, whereas no significant inhibition was observed by SB203580. This extends our previous findings, where 10 mg/kg of U0126 were found to be effective in a rat asthma model [28]. During the preparation of the manuscript it was reported that 30 mg/kg SP600125 inhibited airway eosinophilia in rats [52]. This supports our finding, that targeting JNK reduces allergic airway inflammation.

The inhibitory effect could also be observed by the reduction of cytokine mRNA level. However, the inhibition was not selective for Th2 cytokines. The induction of IFNγ in BAL cells was also inhibited by U0126 and SP600125. This is in line with our findings described above, that costimulation of T cells induces Th1 as well as Th2 cytokines and that MAPK inhibitors are also effective in inhibiting IFNγ. This may be beneficial for the anti-inflammatory effect in the asthma model. It has been shown, that in IFNγ knockout mice less pronounced allergic symptoms such as antigen-specificIgE, eosinophilic infiltration, and airway hyperresponsiveness occur [53]. Therefore, it seems likely that endogenous IFNγ is necessaryfor optimal IgE production also during a secondary response.

The *in vivo *profile of MAPK inhibitors reflects the *in vitro *profile of ICOS costimulated T cells: ERK and JNK inhibitors are effective whereas the p38 inhibitor is not. In contrast, the inhibitory profile of CD28 costimulated T cells is different. There p38 is effective, while the JNK inhibitor is not. In line with this, it has been shown, that ICOS plays an important role in the Th2 effector responses in the lungs, in that inhibition of ICOS suppresses lung inflammation and Th2 cytokine production [54]. Similarly, ICOS-L-Fc augments lung inflammation and the production of cytokines [55]. Together these data indicate the importance of ICOS costimulation in the allergic lung response. Therefore, targeting signaling pathways precipitated by ICOS should dampen the allergic lung inflammation.

Since the three MAPKs are part of different signaling cascades with different upstream and downstream mediators, we asked whether the combination of two MAPK inhibitors may have synergistic effects on the induction of cytokines in CD4+T cells. Our *in vitro *studies did not reveal any such synergistic effect regardless of the combination used. However, we could observe that the simultaneous administration of increasing concentrations of U0126 and 1 μM SB203580 to α-CD3/α-CD28 and α-CD3/α-ICOS stimulated CD4+ T cells led to a right-shift of the dose-response curve for U0126 compared to the administration of U0126 alone. We analyzed this combination also *in vivo*. Whereas treatment of the animals with U0126 alone inhibited late phase eosinophilia markedly as in previous experiments the concomitant treatment with SB203580 diminished the effect of U0126 almost completely. To our knowledge no *in vitro *or *in vivo *studies reported the simultaneous addition of two MAPK inhibitors in T cell activation studies or asthma models. Currently, we investigate the mechanism for this antagonistic action.

## Conclusion

Different MAPK signaling pathways are activated in T cells dependent on the costimulatory receptor engaged. The MAP kinase ERK is important for both stimuli. p38 MAPK activation is important for CD28 induced cytokine synthesis, whereas JNK is important for ICOS induced cytokine synthesis. The different regulation can be exploited to find new specifically targeted drugs aimed for diseases where different costimulatory molecules play an important pathophysiological role. We could demonstrate for the first time that inhibition of the JNK cascade is a therapeutic option for asthma. The specific JNK inhibitor SP 600125 reduced the influx of eosinophils in an animal model of asthma. The development of more specific MEK-ERK and JNK-targeted drugs would support this approach to treat T cell dominated diseases such as asthma.

## List of abbreviations

BAL – bronchoalveolar lavage fluid

ERK – extracellular signal-regulated kinase

ICOS – inducible costimulatory

IL – interleukin

JNK – jun NH2-terminal kinase

MAPK – mitogen-activated protein kinase

OVA – ovalbumine

TCR – T cell receptor

## Authors' contributions

LC carried out ICOS costimulation studies *in vitro *and all *in vivo *studies.

MZ carried out CD28 costimulation studies *in vitro*.

KB participated in study design and coordination.

AP conceived the study, and participated in its design and coordination.

All authors read and approved the final manuscript.

## References

[B1] Busse WW, Lemanske RFJ (2001). Asthma. N Engl J Med.

[B2] Chung KF, Barnes PJ (1999). Cytokines in asthma. Thorax.

[B3] Yssel H, Groux H (2000). Characterization of T cell subpopulations involved in the pathogenesis of asthma and allergic diseases. Int Arch Allergy Immunol.

[B4] Ray A, Cohn L (2000). Altering the Th1/Th2 balance as a therapeutic strategy in asthmatic diseases. Curr Opin Investig Drugs.

[B5] Pahl A, Szelenyi I (2002). Asthma therapy in the new millennium. Inflamm Res.

[B6] Pahl A, Zhang M, Torok K, Kuss H, Friedrich U, Magyar Z, Szekely J, Horvath K, Brune K, Szelenyi I (2002). Anti-inflammatory effects of a cyclosporine receptor-binding compound, D-43787. J Pharmacol Exp Ther.

[B7] Schafer PH, Wadsworth SA, Wang L, Siekierka JJ (1999). p38 alpha mitogen-activated protein kinase is activated by CD28-mediated signaling and is required for IL-4 production by human CD4+CD45RO+ T cells and Th2 effector cells. J Immunol.

[B8] Cefai D, Cai YC, Hu H, Rudd C (1996). CD28 co-stimulatory regimes differ in their dependence on phosphatidylinositol 3-kinase: common co-signals induced by CD80 and CD86. Int Immunol.

[B9] Rudd CE (1996). Upstream-downstream: CD28 cosignaling pathways and T cell function. Immunity.

[B10] Weiss A, Littman DR (1994). Signal transduction by lymphocyte antigen receptors. Cell.

[B11] Bertram EM, Tafuri A, Shahinian A, Chan VS, Hunziker L, Recher M, Ohashi PS, Mak TW, Watts TH (2002). Role of ICOS versus CD28 in antiviral immunity. Eur J Immunol.

[B12] Carreno BM, Collins M (2002). The B7 family of ligands and its receptors: new pathways for costimulation and inhibition of immune responses. Annu Rev Immunol.

[B13] Rudd CE, Schneider H (2003). Unifying concepts in CD28, ICOS and CTLA4 co-receptor signalling. Nat Rev Immunol.

[B14] Tafuri A, Shahinian A, Bladt F, Yoshinaga SK, Jordana M, Wakeham A, Boucher LM, Bouchard D, Chan VS, Duncan G, Odermatt B, Ho A, Itie A, Horan T, Whoriskey JS, Pawson T, Penninger JM, Ohashi PS, Mak TW (2001). ICOS is essential for effective T-helper-cell responses. Nature.

[B15] Coyle AJ, Lehar S, Lloyd C, Tian J, Delaney T, Manning S, Nguyen T, Burwell T, Schneider H, Gonzalo JA, Gosselin M, Owen LR, Rudd CE, Gutierrez-Ramos JC (2000). The CD28-related molecule ICOS is required for effective T cell-dependent immune responses. Immunity.

[B16] Hutloff A, Dittrich AM, Beier KC, Eljaschewitsch B, Kraft R, Anagnostopoulos I, Kroczek RA (1999). ICOS is an inducible T-cell co-stimulator structurally and functionally related to CD28. Nature.

[B17] Yoshinaga SK, Zhang M, Pistillo J, Horan T, Khare SD, Miner K, Sonnenberg M, Boone T, Brankow D, Dai T, Delaney J, Han H, Hui A, Kohno T, Manoukian R, Whoriskey JS, Coccia MA (2000). Characterization of a new human B7-related protein: B7RP-1 is the ligand to the co-stimulatory protein ICOS. Int Immunol.

[B18] Davis RJ (1993). The mitogen-activated protein kinase signal transduction pathway. J Biol Chem.

[B19] Schaeffer HJ, Weber MJ (1999). Mitogen-activated protein kinases: specific messages from ubiquitous messengers. Mol Cell Biol.

[B20] Davis RJ (2000). Signal transduction by the JNK group of MAP kinases. Cell.

[B21] Han J, Ulevitch RJ (1999). Emerging targets for anti-inflammatory therapy. Nat Cell Biol.

[B22] Su B, Jacinto E, Hibi M, Kallunki T, Karin M, Ben-Neriah Y (1994). JNK is involved in signal integration during costimulation of T lymphocytes. Cell.

[B23] Li W, Whaley CD, Mondino A, Mueller DL (1996). Blocked signal transduction to the ERK and JNK protein kinases in anergic CD4+ T cells. Science.

[B24] Nunes JA, Battifora M, Woodgett JR, Truneh A, Olive D, Cantrell DA (1996). CD28 signal transduction pathways. A comparison of B7-1 and B7-2 regulation of the map kinases: ERK2 and Jun kinases. Mol Immunol.

[B25] Matsuda S, Moriguchi T, Koyasu S, Nishida E (1998). T lymphocyte activation signals for interleukin-2 production involve activation of MKK6-p38 and MKK7-SAPK/JNK signaling pathways sensitive to cyclosporin A. J Biol Chem.

[B26] Salmon RA, Foltz IN, Young PR, Schrader JW (1997). The p38 mitogen-activated protein kinase is activated by ligation of the T or B lymphocyte antigen receptors, Fas or CD40, but suppression of kinase activity does not inhibit apoptosis induced by antigen receptors. J Immunol.

[B27] McAdam AJ, Chang TT, Lumelsky AE, Greenfield EA, Boussiotis VA, Duke-Cohan JS, Chernova T, Malenkovich N, Jabs C, Kuchroo VK, Ling V, Collins M, Sharpe AH, Freeman GJ (2000). Mouse inducible costimulatory molecule (ICOS) expression is enhanced by CD28 costimulation and regulates differentiation of CD4+ T cells. J Immunol.

[B28] Pahl A, Zhang M, Kuss H, Szelenyi I, Brune K (2002). Regulation of IL-13 synthesis in human lymphocytes: implications for asthma therapy. Br J Pharmacol.

[B29] Finotto S, De Sanctis GT, Lehr HA, Herz U, Buerke M, Schipp M, Bartsch B, Atreya R, Schmitt E, Galle PR, Renz H, Neurath MF (2001). Treatment of allergic airway inflammation and hyperresponsiveness by antisense-induced local blockade of GATA-3 expression. J Exp Med.

[B30] Myou S, Leff AR, Myo S, Boetticher E, Tong J, Meliton AY, Liu J, Munoz NM, Zhu X (2003). Blockade of inflammation and airway hyperresponsiveness in immune-sensitized mice by dominant-negative phosphoinositide 3-kinase-TAT. J Exp Med.

[B31] Davies SP, Reddy H, Caivano M, Cohen P (2000). Specificity and mechanism of action of some commonly used protein kinase inhibitors. Biochem J.

[B32] Bennett BL, Sasaki DT, Murray BW, O'Leary EC, Sakata ST, Xu W, Leisten JC, Motiwala A, Pierce S, Satoh Y, Bhagwat SS, Manning AM, Anderson DW (2001). SP600125, an anthrapyrazolone inhibitor of Jun N-terminal kinase. Proc Natl Acad Sci U S A.

[B33] Murray BW, Bennett BL, Sasaki DT (2001). Analysis of pharmacologic inhibitors of Jun N-terminal kinases. Methods Enzymol.

[B34] Baxter AG, Hodgkin PD (2002). Activation rules: the two-signal theories of immune activation. Nat Rev Immunol.

[B35] Schwartz RH (2003). T cell anergy. Annu Rev Immunol.

[B36] Manickasingham SP, Anderton SM, Burkhart C, Wraith DC (1998). Qualitative and quantitative effects of CD28/B7-mediated costimulation on naive T cells in vitro. J Immunol.

[B37] Sharpe AH, Freeman GJ (2002). The B7-CD28 superfamily. Nat Rev Immunol.

[B38] Klein-Hessling S, Jha MK, Santner-Nanan B, Berberich-Siebelt F, Baumruker T, Schimpl A, Serfling E (2003). Protein kinase A regulates GATA-3-dependent activation of IL-5 gene expression in Th2 cells. J Immunol.

[B39] Freeman GJ, Long AJ, Iwai Y, Bourque K, Chernova T, Nishimura H, Fitz LJ, Malenkovich N, Okazaki T, Byrne MC, Horton HF, Fouser L, Carter L, Ling V, Bowman MR, Carreno BM, Collins M, Wood CR, Honjo T (2000). Engagement of the PD-1 immunoinhibitory receptor by a novel B7 family member leads to negative regulation of lymphocyte activation. J Exp Med.

[B40] Latchman Y, Wood CR, Chernova T, Chaudhary D, Borde M, Chernova I, Iwai Y, Long AJ, Brown JA, Nunes R, Greenfield EA, Bourque K, Boussiotis VA, Carter LL, Carreno BM, Malenkovich N, Nishimura H, Okazaki T, Honjo T, Sharpe AH, Freeman GJ (2001). PD-L2 is a second ligand for PD-1 and inhibits T cell activation. Nat Immunol.

[B41] Schneider H, Cai YC, Prasad KV, Shoelson SE, Rudd CE (1995). T cell antigen CD28 binds to the GRB-2/SOS complex, regulators of p21ras. Eur J Immunol.

[B42] Tezuka K, Tsuji T, Hirano D, Tamatani T, Sakamaki K, Kobayashi Y, Kamada M (2000). Identification and characterization of rat AILIM/ICOS, a novel T-cell costimulatory molecule, related to the CD28/CTLA4 family. Biochem Biophys Res Commun.

[B43] Koprak S, Staruch MJ, Dumont FJ (1999). A specific inhibitor of the p38 mitogen activated protein kinase affects differentially the production of various cytokines by activated human T cells: dependence on CD28 signaling and preferential inhibition of IL-10 production. Cell Immunol.

[B44] Feito MJ, Vaschetto R, Criado G, Sanchez A, Chiocchetti A, Jimenez-Perianez A, Dianzani U, Portoles P, Rojo JM (2003). Mechanisms of H4/ICOS costimulation: effects on proximal TCR signals and MAP kinase pathways. Eur J Immunol.

[B45] Okamoto N, Tezuka K, Kato M, Abe R, Tsuji T (2003). PI3-kinase and MAP-kinase signaling cascades in AILIM/ICOS- and CD28-costimulated T-cells have distinct functions between cell proliferation and IL-10 production. Biochem Biophys Res Commun.

[B46] Avraham A, Jung S, Samuels Y, Seger R, Ben-Neriah Y (1998). Co-stimulation-dependent activation of a JNK-kinase in T lymphocytes. Eur J Immunol.

[B47] Dong C, Yang DD, Tournier C, Whitmarsh AJ, Xu J, Davis RJ, Flavell RA (2000). JNK is required for effector T-cell function but not for T-cell activation. Nature.

[B48] Parry RV, Rumbley CA, Vandenberghe LH, June CH, Riley JL (2003). CD28 and inducible costimulatory protein Src homology 2 binding domains show distinct regulation of phosphatidylinositol 3-kinase, Bcl-xL, and IL-2 expression in primary human CD4 T lymphocytes. J Immunol.

[B49] Arimura Y, Kato H, Dianzani U, Okamoto T, Kamekura S, Buonfiglio D, Miyoshi-Akiyama T, Uchiyama T, Yagi J (2002). A co-stimulatory molecule on activated T cells, H4/ICOS, delivers specific signals in T(h) cells and regulates their responses. Int Immunol.

[B50] Escott KJ, Belvisi MG, Birrell MA, Webber SE, Foster ML, Sargent CA (2000). Effect of the p38 kinase inhibitor, SB 203580, on allergic airway inflammation in the rat. Br J Pharmacol.

[B51] Underwood DC, Osborn RR, Kotzer CJ, Adams JL, Lee JC, Webb EF, Carpenter DC, Bochnowicz S, Thomas HC, Hay DW, Griswold DE (2000). SB 239063, a potent p38 MAP kinase inhibitor, reduces inflammatory cytokine production, airways eosinophil infiltration, and persistence. J Pharmacol Exp Ther.

[B52] Eynott PR, Nath P, Leung SY, Adcock IM, Bennett BL, Chung KF (2003). Allergen-induced inflammation and airway epithelial and smooth muscle cell proliferation: role of Jun N-terminal kinase. Br J Pharmacol.

[B53] Hofstra CL, Van Ark I, Hofman G, Nijkamp FP, Jardieu PM, Van Oosterhout AJ (1998). Differential effects of endogenous and exogenous interferon-gamma on immunoglobulin E, cellular infiltration, and airway responsiveness in a murine model of allergic asthma. Am J Respir Cell Mol Biol.

[B54] Gonzalo JA, Tian J, Delaney T, Corcoran J, Rottman JB, Lora J, Al-garawi A, Kroczek R, Gutierrez-Ramos JC, Coyle AJ (2001). ICOS is critical for T helper cell-mediated lung mucosal inflammatory responses. Nat Immunol.

[B55] Guo J, Stolina M, Bready JV, Yin S, Horan T, Yoshinaga SK, Senaldi G (2001). Stimulatory effects of B7-related protein-1 on cellular and humoral immune responses in mice. J Immunol.

